# Nest location and architecture as primary drivers of variation in UV reflectance in avian eggs

**DOI:** 10.1098/rspb.2025.0180

**Published:** 2025-05-21

**Authors:** Maria Elisa Mendiwelso, Carlos Daniel Cadena, David Ocampo

**Affiliations:** ^1^ Departamento de Ciencias Biológicas, Facultad de Ciencias, Universidad de los Andes, Bogotá, Colombia; ^2^ Department of Ecology and Evolutionary Biology, Princeton University, Princeton, NJ 08544, USA

**Keywords:** eggshell colour, light exposure, nest, macroecology, UV reflectance, egg

## Abstract

Two main hypotheses have been proposed to explain the function of ultraviolet (UV) reflection in avian eggs. The UV resistance hypothesis suggests that high UV reflectance protects embryos against solar exposure in open nests, whereas the egg detectability hypothesis posits that higher UV reflectance helps eggs stand out against the dark background of the nest, making them easier for parents to locate in enclosed nests. Therefore, eggshell reflection in the UV spectrum may serve multiple (possibly even opposing) forces, including UV protection and visual signalling. We tested these two hypotheses using large-scale comparative analyses of eggshell UV reflection for over 500 avian species while considering the influence of various ecological, life history and environmental traits associated with light exposure. We did not find strong support for either of the two hypotheses across all birds. However, in two clades exhibiting notably high levels of UV reflectance (Passeriformes and Charadriiformes), species with higher UV reflectance values predominantly nest in open nests, suggesting a stronger effect of the UV resistance hypothesis. This research contributes to a deeper understanding of the mechanisms driving UV coloration in avian eggs and unravels the interplay between life history traits potentially associated with UV reflectance in specific clades under variable nesting conditions.

## 1. Introduction

Although most species of birds exhibit some degree of colouring in their eggshells, avian eggs are highly variable in appearance. This has raised interest from various evolutionary [1], behavioural [2], morphological [3,4] and ecological perspectives [5]. Egg colours range from pure white to a spectrum of blue–green and brown hues produced by pigments and serve multiple functions in avian reproduction [6,7]. Such functions include decreasing predation risk [8], egg mimicry in avian brood parasites [9], thermal regulation [10], camouflage [11] and even sexual signalling [12].

In addition to overall coloration, ultraviolet (UV) reflectance is another crucial aspect of egg coloration recently garnering attention for its potential impacts on predation [8], parental investment [13] and photoprotection [10]. Because UV solar radiation, particularly in the 290−320 nm range, poses a risk during embryonic development [14], eggshell colour may protect the embryo by reducing UV transmittance across the eggshell [15]. However, the colour of avian eggshells interacts with solar radiation in a complex way, such that more intense coloration (i.e. with more pigment) may protect the developing embryo from detrimental effects caused by UV rays, but intense colours may also cause overheating [5,10]. Accordingly, the UV resistance hypothesis predicts that egg coloration should be related to light exposure patterns and intensity, a relationship which might be mediated by other environmental variables and species traits. Under this hypothesis, more exposed eggs would be more likely to be damaged by solar radiation, and this selective pressure should result in adaptations in eggshell coloration to reduce harm to the embryo. Bearing in mind that exposure to light is influenced by various factors, including the nest site and structure, vegetation, incubation patterns and other parental behaviour, as well as local environmental conditions [10,16], one would expect that eggs laid at more exposed sites (e.g. those laid in open-cup nests or on open platform nests on the ground), in open habitats and at higher latitudes, exhibit greater UV reflectance [17].

Alternative adaptive hypotheses for the role of UV reflection driving egg coloration in different nesting environments often focus on signalling cues linked to selective pressures exerted by nest predators or brood parasites [5,18,19]. Other hypotheses focus on signalling cues, related to how detectable eggs are by their parents, predicting patterns opposite to those expected under the UV resistance hypothesis. Because many birds possess photoreceptors sensitive to UV light [20], egg coloration in the non-visible spectrum for humans (i.e. <380 nm) may have been selected to enhance the detectability of eggs because UV colours would stand out against the dark background of the nest, making them easier for parents to locate [21]. This egg detectability hypothesis predicts greater UV reflectance in birds nesting in darker environments [22].

In sum, eggshell reflection may serve multiple, possibly opposing functions, including UV protection and visual signalling, depending on the environmental context and various life-history traits of species (table 1). The interplay among these factors may thus explain the variation in eggshell UV colour among species and populations, as well as the evolutionary transitions between different egg coloration phenotypes. Despite its potential significance, comparative research on the variation of UV reflectance in bird eggs remains limited, with a predominant focus on elucidating the function and underlying structure of UV reflectance in particular species and not on exploring variation across different phylogenetic lineages. Here, we use a phylogenetic comparative approach across a broad diversity of birds at a global scale (approx. 510 species representing 90% of avian orders) to test the UV-resistance hypothesis and the egg detectability hypotheses posed to explain patterns in UV reflectance while considering the influence of various ecological, life history and environmental traits associated with light exposure.

**Table 1.** Trait definitions and hypotheses about the potential ecological, life history and environmental correlates of eggshell UV reflectance.

	trait	definition of variable	hypotheses	key reference
ecological	nest exposition	**open**: nests that are rounded, with a central depression and no roof **domed**: enclosed nest with a roof constructed by a bird **cavity**: nesting structure that is excavated or naturally occurring (i.e. cave, tree hole, human-enclosed structure)	in open nests, higher UV reflectance is expected as a form of protection against UV rays	[5,10]
			in globular and cavity nests, it is expected that eggs will have higher UV reflectance in order to be detected more easily by parents	[22,23]
	habitat density	**dense habitats**: species primarily lives in the lower or middle storey of forest, or in dense thickets, dense shrubland, etc **semi-open habitats**: species primarily lives in open shrubland, scattered bushes, parkland, low dry or deciduous forest, thorn forest **open habitats**: species primarily lives in desert, grassland, open water, low shrubs, rocky habitats, seashores, cities; also applies to species living mainly on top of forest canopy (i.e. mostly in the open)	in more open habitats, eggs will reflect more UV because there is less vegetation to provide shade, making the eggs more exposed to solar radiation and therefore requiring more reflection of these potentially harmful waves for the egg and the developing embryo inside	[24,25]
phenotypic	egg coloration	average brightness	even a highly reflective white egg does not reflect all light, so some of that light must be absorbed by pigments to prevent transmission to the embryo; therefore, eggs with pigmentation, especially biliverdin, are expected to reflect more UV than immaculate eggs	[26]
			because unpigmented (immaculate) eggs are often found in closed nests (such as cavities) and they reflect more UV, this is expected to serve as a signalling pathway to parents	[10,27]
	egg shape	egg shape index	eggs with greater length will reflect more UV because they have a larger surface area of the egg exposed to radiation, unlike more oval-shaped eggs	[28]
environmental	central latitude	centroid latitude (decimal degrees)	UV radiation levels are higher near the equator, so eggs are expected to reflect more UV closer to the equator	[15]

## 2. Methodology

### (a) Study system and data collection

#### (i) Study system

Our study involved measuring over 1000 eggs from various individual birds of 510 species, covering approximately 90% of all bird orders from around the world, including a substantial number of eggs from tropical regions (electronic supplementary material, table S1). Eggs were studied in the Cornelis J. Marinkelle Oology Collection at the Instituto Humboldt (IAvH-CJM) located in Villa de Leyva, Boyacá, Colombia. We aimed to include at least two eggs from each species from different clutches and then averaged the values for each species.

#### (ii) Eggshell UV reflectance

Reflectance spectra were taken for the standardized visible wavelength range for UV-sensitive songbirds (Passeriformes; 320−700 nm). We used a fibre optic spectrophotometer (Ocean Optics USB2000+UV VIS) with a light source (Ocean Optics PX-2) and a 400 µm reflection probe (Ocean Optics R400-7) held perpendicular to the eggshell (90°), which emits light across a wavelength range of approximately 220−750 nm. This setup allows partial coverage of the UV spectrum, which spans wavelengths from 100 to 400 nm and is traditionally subdivided into three regions: UVC (100–280 nm), UVB (280–315 nm) and UVA (315–400 nm). Our equipment captures the UVA and UVB ranges, and only partially the UVC range, as wavelengths below 220 nm are not emitted by the PX-2 light source. To account for variation within each egg, we took three measures per egg: the blunt end, the equator and the pointed end [5]. In eggs with spots distributed throughout the shell, we took additional measurements in the spots in the three regions, resulting in six measurements per maculated egg [5]. We then calculated UV-chroma using the summary function of R [29] in package PAVO [30] as a proportion of UV-reflectance from total reflectance (R300−400/R300−700, S1U) for each egg [31], followed by normalization setting maximum reflectance to 1, and then calculated the mean of the UV reflectance measurements per species.

**Figure 1.**
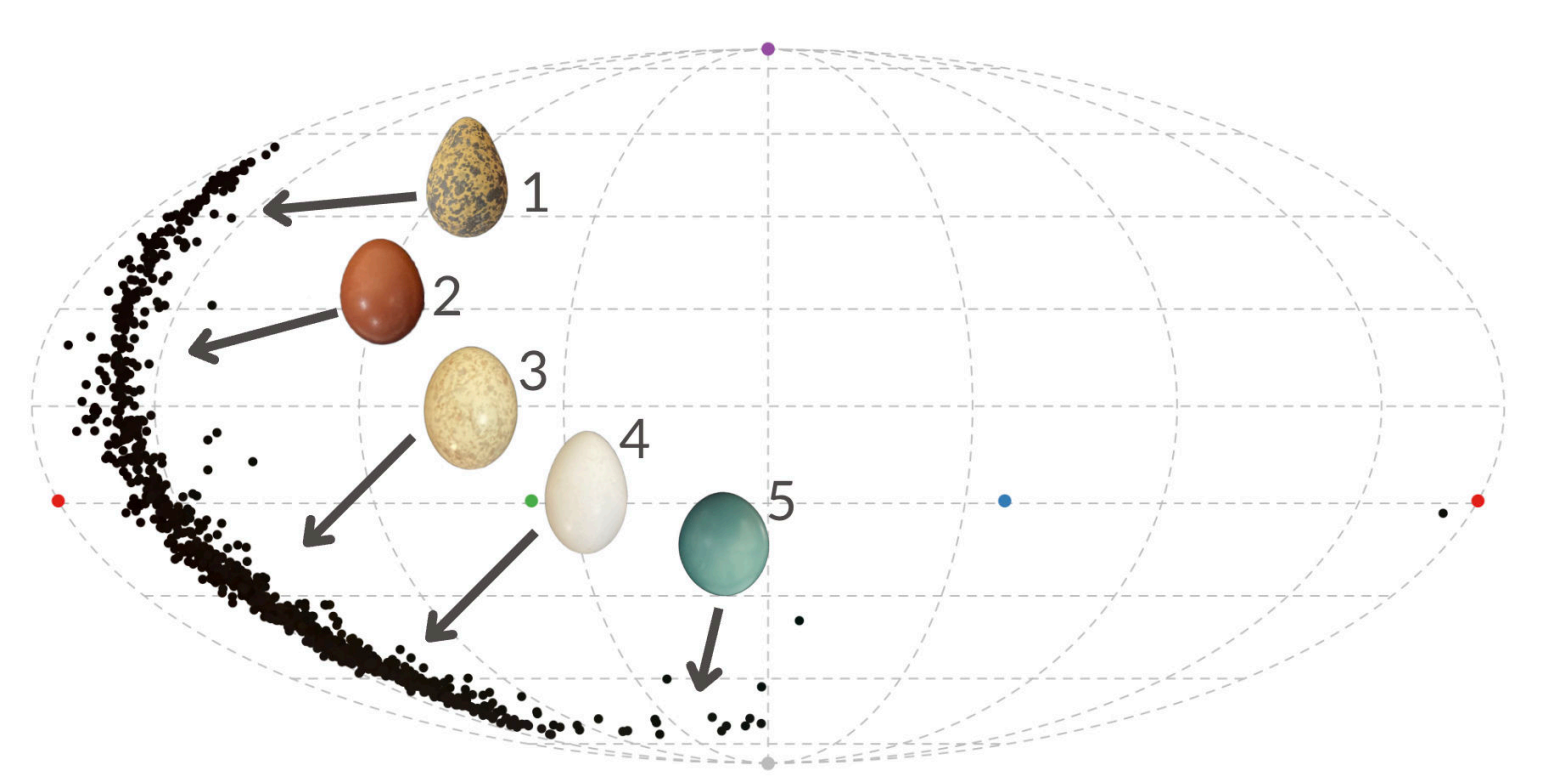
Mollweide projection of the hue distribution of natural eggshell colour within the ultraviolet-sensitive avian colour space, with five representative eggs: (1) *Chlidonias niger*, (2) *Horornis diphone*, (3) *Nyctidromus albicollis,* (4) *Melanerpes carolinus* and (5) *Tinamus major*. Photos: Maria Elisa Mendiwelso.

To explore variation in eggshell colour, we projected the spectral data into an avian ultraviolet-sensitive colour space using a tetrahedral colour model (hereafter ‘natural eggshell colours’) (figure 1) [32]. Additionally, variation in UV reflectance across avian orders was examined using a Kruskal–Wallis test.

#### (iii) Ecological, life-history and environmental factors associated with UV reflectance

We identified ecological, life-history and environmental traits that may correlate with UV reflection in eggs (table 1). We then searched for data on the degree to which eggs may be exposed to light for each species based mainly on Birds of the World [33], supplemented with primary literature. To test for possible associations between UV reflectance and species traits, we compiled data on nest exposure [22,34], habitat density [15] and latitude [15] for each species. We also recorded egg maculation (absence/presence) [10,26,27,35] and egg size [24].

We compiled nest descriptions for 510 species, encompassing 33 orders (electronic supplementary material, table S1). Each species was assigned to one of three primary nest exposure categories: open nests (i.e. rounded nests with a central depression and no roof*, n* = 350), domed nests (i.e*.* globular structures*, n* = 68) and cavity nests (i.e. nests built inside a tree cavity, cave or burrow, *n* = 92; [24,36]). For each species, we also assigned one of the three habitat types: dense (forest, thickets, dense shrubland), semi-open (open shrubland, scattered bushes, parkland, low dry or deciduous forest) or open (deserts, grassland, low shrubs, rocky habitats, seashores, cities, forest canopy) [37]. To characterize geographic distributions, we also recorded the latitude centroid, i.e. the geometric centre of the species breeding range [37].

For each egg, we described colour using the average brightness taken from the Pavo summary function, which provides mean spectral reflectance from 400 to 700 nm. This variable is normalized to a range from 0 to 1, where higher values of mean brightness indicate a lighter colour (i.e. white or cream), whereas lower values indicate a darker colour (i.e. brown or red). For each of the eggs, we took photographs using a reference scale with a precision of 0.1 mm. An egg shape index was calculated as the egg’s width divided by its length (mm) as measured in the software ImageJ [38], a simple but well-known metric indicating whether an egg is more oval (values close to 1) or elongated (values close to 0 [28,39]; see electronic supplementary material, table S2 for complete dataset).

### (b) Phylogenetic comparative methods

For the 510 species for which we collected colourimetric, ecological, phenotypic and environmental data, we downloaded a 1000-tree subset of the bird species in our data set from Birdtree [40]. Although this phylogeny has limitations in sampling and resolution, we believe such limitations are unlikely to obscure the broad-scale patterns we investigated. The tree subsets were taken from Ericson *et al*. [41]. We then employed 1000 randomly sampled phylogenies and used TreeAnnotator [42] to obtain a maximum clade-credibility tree, which we used as the species-level phylogeny for comparative analyses. To examine the evidence for phylogenetic signal in eggshell traits, we employed the ‘phylosig’ function from the Phytools R package [43] to compute Pagel’s lambda. We then used the ‘fastAnc’ and ‘contMap’ functions, both implemented in Phytools [43], to estimate and visualize ancestral states for eggshell UV coloration (95% CI) across the tree using maximum likelihood.

Then, to evaluate the relative importance of our ecological, phenotypic and environmental predictors on eggshell UV reflectance to test our predictions for the UV resistance and the egg detectability hypotheses (table 1) [44,45], we employed a generalized linear model framework using the *glmulti* function in R. This methodology facilitated the identification of the most relevant variables and model combinations (electronic supplementary material, figure S2; table 1). Among the variables, *brightness*, *nest exposure* and *habitat density* exceeded the significance threshold, indicating their prominence within our model. Subsequently, we utilized the *dredge* function from the MuMIn package [46] to generate a comprehensive set of models incorporating the most pertinent covariates identified in the prior analysis [47]. Because there was a strong phylogenetic signal in UV reflectance, we used phylogenetic generalized least squares (PGLS) implemented in the R package ‘caper’ [48] to test for interactions between the most relevant variables associated with light exposure (shown in table 2) and eggshell UV reflectance on the best model.

**Table 2.** Comparison of phylogenetic generalized least squares models explaining the eggshell UV chroma reflection of 510 species of birds used in the study. Lower δAIC scores (AIC model (i) – AIC best model) indicate better models. Only models that denote the top-ranked models (those with ΔAIC ≤ 0.15) were shown.

model	df	AIC	ΔAIC	W
brightness × nest exposition	6	−1118.30	0	1.000
brightness × nest exposition × central latitude	12	−1114.70	0.000	0.062
egg shape + nest exposition	4	−1080.90	0.00	0.128
egg shape + nest exposition + central latitude	5	−1078.90	0.00	0.034
nest exposition × central latitude	6	−1077.21	0.03	0.028
brightness + habitat density	4	−1091.43	0.07	0.239
brightness + egg shape + habitat density	5	−1091.60	0.10	0.121
brightness × habitat density	4	−1091.50	0.10	0.985
egg shape + central latitude	3	−1081.00	0.10	0.131
nest exposition	3	−1080.90	0.10	0.248
habitat density	3	−1080.80	0.10	0.235
egg shape + habitat density	4	−1080.50	0.10	0.106
habitat density + central latitude	4	−1078.80	0.10	0.063
egg shape + nest exposition + habitat density	6	−1078.20	0.10	0.025
egg shape + nest exposition + habitat density + central latitude	7	−1076.10	0.10	0.007
nest exposition + central latitude	4	−1078.90	0.15	0.066

Building on our understanding of the most influential variables on UV reflectance as identified in our best model—namely brightness, nest exposure and habitat density, which surpassed the significance threshold—we aimed to determine whether the UV resistance or the egg detectability hypothesis provides a better explanation for UV reflectance. To investigate this, we conducted a phylogenetic ANOVA using the Geiger package, considering different nesting strategies (open, closed and domed) and habitat types (open, semi-open and closed). Subsequently, post hoc analyses were performed to further elucidate the relationships between the selected predictor variables and UV reflectance.

## 3. Results

### (a) Taxonomic distribution of UV reflectance

We found substantial variability among birds in the distribution of natural eggshell colours within the tetrahedral colour space of ultraviolet-sensitive avian vision, reflected in differences in luminance, chroma and the angular coordinates (theta and phi) that describe hue (figure 1). This prompted us to explore how this colour variation, focusing on UV reflectance, was distributed within and among different avian orders. UV reflectance in eggs varied significantly among avian orders (*χ*^2^(32) = 148.070, *p* < 0.001); those with the highest mean UV reflectance were Casuariiformes, Passeriformes and Charadriiformes, whereas those with the lowest were Struthioniformes, Coliiformes and Musophagiformes (electronic supplementary material, figure S1). Within some orders, UV reflectance was homogeneous, whereas in others it exhibited substantial variation among species, possibly attributable to variation in nesting behaviour. For instance, most species of Piciformes excavate nest cavities in trees, and all had very similar values of UV reflectance (mean = 0.081 ± s.d. = 0.024), whereas species of Caprimulgiformes may nest in varied microenvironments, including ground and branches of semi-open habitats (Caprimulgidae, Trochilidae) or in dark caves or under eaves of bridges and buildings (Apodidae, Steatornithidae) and have more variation in UV reflectance (mean = 0.126 ± s.d. = 0.059).

### (b) Ancestral character state reconstruction

**Figure 2.**
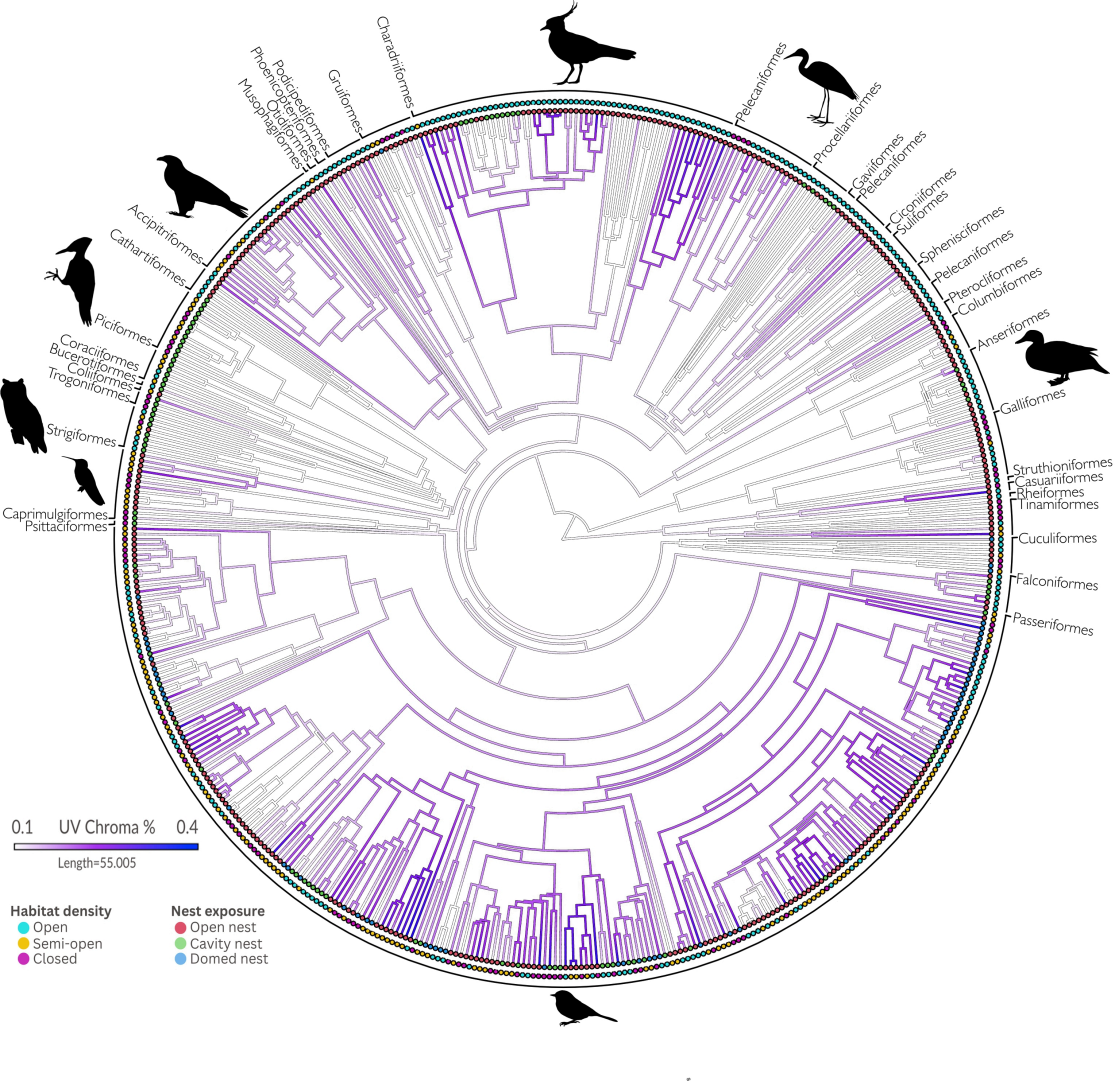
Maximum-likelihood estimates of ancestral states of the percentage of UV chroma reflected by avian eggshells. The scale bar indicates UV chroma from low values (whiter colours) to high values (purple colours). Changes along branches are calculated by interpolation [43]. The two sets of coloured circle symbol matrices at the end of each branch tip, the innermost circle corresponding to nest exposition and the outermost symbol corresponding to habitat density. We follow the taxonomy proposed by Clements *et al*. [49].

Based on our ancestral character state reconstructions, the most recent ancestor of birds probably had comparatively low levels of UV reflectance in its eggshell (0.103 [−0.029, 0.234] 95% CI; figure 2). From such a state, greater UV reflectance evolved independently multiple times across various orders and families. However, increases in UV reflectance appear to have been particularly concentrated in some clades, such as the oscine passerines, some families of Charadriiformes (Charadriidae, Laridae, Stercorariidae and Trunicidae) and independently in some species, such as *Podilymbus podiceps* (Podicipedidae), *Harpactes erythrocephalus* (Trogonidae) and *Chordeiles acutipennis* (Caprimulgidae; figure 2). Despite such changes, UV reflectance was evolutionarily conserved across the tree (*λ* = 0.766, LR test *p* < 0.001).

### (c) UV reflectance and covariates of light exposure

**Figure 3.**
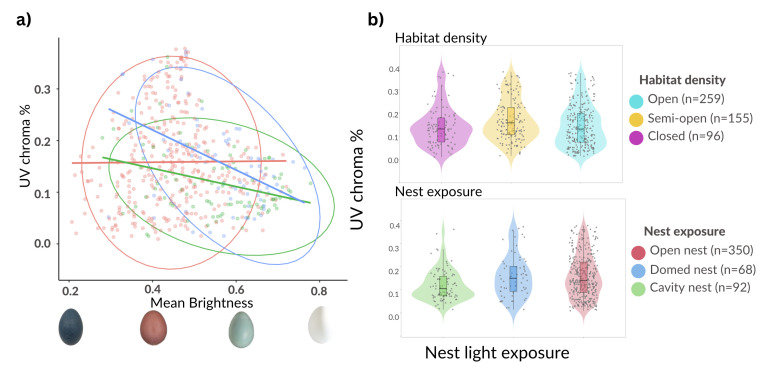
(a) Scatter plot illustrating the interaction between UV reflection and mean brightness, with each point representing a species, grouped by nest exposure group (open, domed, cavity) using ellipses. Eggs placed in open and domed nest expositions had lower mean brightness and higher UV chroma compared with cavity nests. (b) Violin plot distribution of nest exposure and habitat density on a scale ranging from least exposed to most exposed, considering the mean value for each category.

Prior to selecting our optimal model, we sought to examine the model-averaged importance of variables predicting UV reflectance. The most important variables were egg brightness, nest exposure and habitat (electronic supplementary material, figure S2). Four out of the five predictor variables (i.e. egg brightness, egg shape, nest exposure and latitude) were present in the set of best models explaining UV reflectance (i.e. models with ΔAIC = 0; table 2). The top-ranked model incorporated the interaction between brightness and nest exposure (table 3), and distinct patterns were observed among the three different nest exposure types. In open nests, which represent the predominant category of nest exposure in our dataset (*n* = 350), we observed substantial variation in UV reflectance and egg brightness. Conversely, cavity nesters displayed lower levels of UV reflectance compared to those of open and domed nests. We found a significant interaction between nest brightness and nest type through the PGLS analysis (*t*-value = −5.7289, *p* < 0.001), whereby there was a negative correlation between egg brightness and UV reflectance only in domed nests (figure 3a). This category included nests built with a variety of materials (e.g. mud in the rufous hornero *Furnarius rufus*; sticks in the ringed antpipit *Corythopis torquatus*), meaning that the degree to which light enters nests in this category can be quite variable. This could explain variation in UV reflectance and its relationship with egg colour only for this nest type and not for others, in which light exposure appears more uniform across species (figure 3a).

**Table 3.** Summary results of the PGLS analyses. Association between eggshell UV chroma and nesting ecology variables in the top mode in 510 species of birds.

UV variable	estimate	unconditional SE	95% **CI**	relative importance
intercept	0.101498	0.066762	(−0.029, 0.232)	
brightness	0.007908	0.042673	(−0.076, 0.092)	0.995
nest exposure				0.248
domed	0.187961	0.037434	(0.115, 0.261)	
cavity	0.034456	0.040990	(−0.046, 0.115)	
brightness: nest exposure				1
brightness: domed	−0.395873	0.069352	(−0.532, −0.260)	
brightness: cavity	−0.081357	0.078899	(−0.236, 0.073)	

Note: open nest was the reference category.

### (d) UV reflection in relation to nest exposure

We did not uncover a significant effect of habitat density (phylogenetic ANOVA, *F* = 4.628, *p* = 0.457) nor nest type (phylogenetic ANOVA, *F* = 7.337, *p* = 0.406, all post hoc comparisons with Holm correction *p* > 0.05) on UV reflectance of eggs, meaning that there was no support for either of our main hypotheses relating coloration to light exposure (i.e. UV resistance and egg detectability). Nonetheless, we observed a trend for a slight increase in UV chroma with increasing nest light exposure (figure 3b). This pattern would align with the predictions of the UV resistance hypothesis that eggs at exposed locations (e.g. open cup nests or ground-placed open platform nests) would exhibit heightened UV reflectance.

We observed increased UV reflectance on the oscine passerine clade, which may be explained by the significant diversity in nest types, including open, closed and cavity nests. However, despite this variety, eggs exhibiting higher UV reflectance were generally those of species constructing open nests (figure 2). Charadriiformes, unlike passerine birds, predominantly nest on the ground and in exposed environments; a large proportion of species in this order exhibit high UV reflectance (figure 2).

## 4. Discussion

Birds represent the only living amniotes with coloured eggs, which have long been considered a unique avian innovation [26,35]. Despite attempts to comprehend the variation in eggshell coloration among bird species [1,28], and to uncover the structural and non-structural functions of egg coloration [1,35], the potential role of UV coloration reflected by eggshells (which is perceived by many bird species) and its variation across different taxonomic groups remain largely unexplored. Our study is the first broad-scale macro-ecological investigation of UV coloration in avian eggshells in a phylogenetic framework, encompassing a diverse global sample for over 500 avian species. Our results contribute to a deeper understanding of the mechanisms driving UV coloration in avian eggs and shed light on the complex interplay between selection pressures and the evolution of UV coloration in bird eggs.

The egg detectability hypothesis and the UV resistance hypothesis make contrasting predictions regarding UV reflectance in bird eggshells. According to the egg detectability hypothesis, eggs in nests with limited light exposure, such as domed or cavity nests situated in dense habitats and higher latitudes, should exhibit greater UV reflectance [22]. Conversely, the UV resistance hypothesis suggests that eggs in nests with higher light exposure, such as those in open or semi-open habitats and lower latitudes, should display increased UV reflectance [5,10]. Our study yielded two key findings relevant to gauging support for these alternative hypotheses. First, egg brightness, nest exposure and habitat density emerged as the most influential variables associated with UV reflectance by eggs. The prominence of these variables is critical for evaluating the validity of the hypotheses, as they reflect the ecological conditions that affect UV exposure. Specifically, if the data support the egg detectability hypothesis, we would expect higher UV reflectance in eggs from nests with limited light exposure [22,23]; conversely, if the UV resistance hypothesis holds, we would see increased reflectance in eggs from well-lit environments [5,10]. Second, upon testing whether the observed patterns of UV reflectance could be attributed to either the UV resistance or the egg detectability hypotheses, we found no statistically significant influence of nest type or habitat density—two variables that most effectively describe light exposure—on UV reflectance across all avian taxa examined. Nevertheless, a closer examination of specific clades revealed that our findings lend stronger support to the UV resistance hypothesis within Passeriformes and Charadriiformes, wherein most species nesting in open environments exhibited heightened UV reflectance values. Moreover, a trend emerged indicating that mean UV reflectance across all species exhibited a slight—albeit non-significant—increase with greater light exposure of the nest, further reinforcing the notion that ecological light conditions partly shape UV reflectance patterns in bird eggs.

Based on our best model, our findings indicate a complex relationship between egg coloration and UV reflectance, suggesting that the evolutionary pressures influencing avian egg pigmentation might extend beyond simple light reflection. The significant interaction observed between brightness and nest exposure highlights the importance of ecological context in shaping UV reflectance. Notably, the substantial variation in UV reflectance within open nests could imply that eggs in these environments are under different selective pressures compared to those in cavity or domed nests [50]. Our findings revealed that the ancestral avian egg exhibited relatively low levels of UV reflectance, which is often associated with darker coloration, given that white is expected to reflect all colours, including the UVA range, compared to pigmented eggs [51]. This suggests that early bird eggs may have evolved under ecological conditions where UV protection was less critical, possibly because some species may behaviourally adjust exposure through high nest attendance or because some of the earliest dinosaur ancestors of birds buried their eggs underground and covered them with substrate [52]. However, even highly reflective white eggs do not reflect all light, and pigmentation might be adaptive if it blocks damaging light wavelengths from entering the egg [10].

We focus on two key pigments that might be adaptive if they block damaging light wavelengths from entering the egg: biliverdin and protoporphyrin IX. Biliverdin—the pigment responsible for blue and green coloration—confers higher reflectance in the UV range compared to protoporphyrin IX—the pigment responsible for red and brown coloration, which is less effective at blocking UV transmittance [32,53]. This might lead one to expect that eggs with different colours would reflect UV light differentially. However, in our dataset, eggs from a wide spectrum of visible coloration (i.e. brightness) exhibited similar UV reflectance, implying that the relationship between visible coloration and UV reflectance is still unclear. Considering that pigment is costly to produce [54], species in which reducing the effects of UV radiation on eggs is adaptive but is not achieved via egg colour may instead reduce exposure by other mechanisms, such as incubation behaviour [27].

Two main clades exhibited notably high levels of UV reflectance. The first clade involves passerines, in which most species with open nests had high UV reflectance that can be attributed to the extensive diversity in breeding strategies, which arise due to varying survival requisites dependent on their respective habitats [55,56]. The second clade comprises the Charadriiformes. Given that these species typically lay cryptically coloured eggs in open environments with consistently high light exposure, it is reasonable to infer that eggshell UV reflection and pigmentation may function as protective mechanisms for developing embryos. However, other factors, such as parental behaviour—including factors like incubation period [57]—as well as colour matching between eggshell ground colour and nest background colour, may also play a supplementary role in embryo protection [58]. It is plausible, therefore, that high UV reflectance in eggs serves as an additional layer of protection, alongside ecological and breeding factors, representing an adaptation to counteract transmittance through the eggshell and prevent overheating via absorption. This would suggest support for the UV resistance hypothesis [10]. However, further research on the reproductive biology of species of Charadriiformes is crucial to explore how considering other selective pressures (e.g. camouflage [59]) may help one better elucidate the evolution of UV eggshell colour [5].

The egg detectability hypothesis predicts that eggs laid in closed nests have higher UV reflectance to increase the detectability of eggs by adults in dark nesting environments [22]. This was only partially supported in our study because we found that eggs laid in domed nests— but not those in natural cavities—had higher UV reflectance compared to other nest types and locations. This could suggest that birds constructing dome-shaped nests, which are characterized by greater light exposure than cavity nests, significantly rely on nest luminance to locate their own eggs or to identify parasitic eggs [23,60]. Given that no visual system can function effectively in complete darkness, certain species may lack the requisite visual sensitivity to detect UV coloration in the low-light conditions typical of cavity nests [61]. This limitation may explain the diminished UV reflectance observed in eggs laid within these darker nesting environments compared to those situated in more illuminated dome-shaped nests. The lack of broader statistical support for the egg detectability hypothesis could also reflect methodological limitations. For instance, nest type was categorized based on general descriptions, which may not accurately capture the spectrum of variation in internal luminance across different nesting environments. Despite these limitations, our findings underscore the potential relevance of nest luminance and visual ecology in shaping egg coloration. Future studies incorporating finer-scale light measurements and visual system data across taxa, along with the most robust DNA-based phylogenies for the focal clades, could provide further insights into the adaptive role of UV reflectance in closed nesting environments.

As for the UV resistance hypothesis, the selective implications of solar radiation for eggs and their pigments depend on the link between reflectance, transmittance and absorbance [10]. Different pigments have different properties and can affect the eggshell in multiple ways, with egg colours in principle having possibly opposite effects, either acting as a parasol or causing a dark-car effect [10]. In our best model, we observed a negative correlation between egg brightness and nest exposure. Given that lower brightness values—indicating darker colours—were associated with higher UV reflectance, we suggest that higher UV reflectance may both modulate heat that enters the egg and prevent harmful UV transmittance throughout the eggshell in open nests, as proposed in the UV resistance hypothesis. To better understand the validity and generality of the UV resistance hypothesis, evaluating other features that may prevent harmful effects of UV transmittance (e.g. the microstructure of the eggshell cuticle [31]) would be of interest.

Finally, although this study encompassed a diverse array of bird species in investigating the potential function of UV reflectance in avian eggs, many of the species for which we gathered egg colour data could not be included in comparative analyses due to insufficient information about the nest site and location. This gap persists because our understanding of the breeding biology of numerous bird species remains incomplete [62]. Detailed field-based studies, rooted in natural history, are indispensable for collecting data on factors such as incubation period, clutch size and parental behaviours related to nest care, which are crucial for comprehending the role of UV reflection across the entire spectrum of avian diversity. This study establishes the groundwork for future analyses focused on specific clades to unravel the interplay between factors potentially associated with UV reflectance. In future work, it would also be valuable to investigate the selective pressures and potential combined effects influencing UV reflectance, such as predation, parasitism and physiological constraints.

## Data Availability

All data supporting the results of this study are publicly accessible in Figshare for both statistical analysis [63] and supplementary material [64,65].
